# Event-Related Potential Evidence of Implicit Metric Structure during Silent Reading

**DOI:** 10.3390/brainsci9080192

**Published:** 2019-08-08

**Authors:** Mara Breen, Ahren B. Fitzroy, Michelle Oraa Ali

**Affiliations:** 1 Department of Psychology and Education, Mount Holyoke College, South Hadley, MA 01075, USA; 2 Department of Psychological and Brain Sciences, University of Massachusetts, Amherst, MA 01003, USA; 3 Department of Cognitive, Linguistic, and Psychological Sciences, Brown University, Providence, RI 02912, USA

**Keywords:** implicit prosody, reading, meter, rhythm, lexical stress, event-related potentials, poetry

## Abstract

Under the Implicit Prosody Hypothesis, readers generate prosodic structures during silent reading that can direct their real-time interpretations of the text. In the current study, we investigated the processing of implicit meter by recording event-related potentials (ERPs) while participants read a series of 160 rhyming couplets, where the rhyme target was always a stress-alternating noun–verb homograph (e.g., permit, which is pronounced PERmit as a noun and perMIT as a verb). The target had a strong–weak or weak–strong stress pattern, which was either consistent or inconsistent with the stress expectation generated by the couplet. Inconsistent strong–weak targets elicited negativities between 80–155 ms and 325–375 ms relative to consistent strong–weak targets; inconsistent weak–strong targets elicited a positivity between 365–435 ms relative to consistent weak–strong targets. These results are largely consistent with effects of metric violations during listening, demonstrating that implicit prosodic representations are similar to explicit prosodic representations.

## 1. Introduction

According to the Implicit Prosody Hypothesis [1,2,3], readers generate imagined representations of prosodic structure during silent reading that are similar to the explicit prosodic representations that readers produce when reading aloud. This hypothesis has been supported by behavioral evidence demonstrating similarity between real and imagined representations of a variety of prosodic phenomena, including intonation, phrasing, stress, and meter [4,5]. For example, evidence for implicit intonational structure is provided by the fact that readers are faster to recognize target words that are produced aloud with a previously imagined intonation contour [6,7]. Readers impose implicit phrase boundaries in sentences that are long enough to have a phrase break [8] and tend to balance the size of adjacent phrases even during silent reading [9,10], providing evidence for implicit prosodic phrasing. Readers take longer to silently read words with two stressed syllables than words with one stressed syllable [11], and take longer to read sentences in which a local lexical stress pattern mismatches the predicted metric structure as determined by prior sentence material [12,13,14,15,16], providing evidence for an implicit metric structure. Although these behavioral similarities between patterns associated with explicit and implicit prosody provide indirect support for implicit prosodic representations, they cannot tell us to what extent implicit prosodic representations are processed similarly to explicit prosodic representations. In the current study, we used event-related potentials (ERPs) to investigate the processing of implicit prosodic representations, and how it compares to that of explicit prosody.

### 1.1. Behavioral Studies of Explicit and Implicit Linguistic Metric Representation

The specific focus of the current study is the similarity between implicit and explicit metric processing. For this investigation, we exploit metrical regularity in English; English is a stress-timed language, meaning that speakers produce temporally regularized sequences of strong (stressed) and weak (unstressed) syllables. The metric structure in stress-timed languages is conveyed by the timing of strong syllables [17,18]. There are constraints on the ordering of strong and weak beats in stress-timed languages, as strong beats tend to occur at regular intervals [19], speakers avoid clashes of strong beats and lapses of weak beats [20], and under some circumstances, speakers shift the location of stress on words to maintain metric regularity (e.g., thirTEEN MEN → THIRteen MEN) [18]. Speakers signal strong syllables in speech with a variety of acoustic cues, including longer duration and higher intensity [21,22,23,24]. Strong syllables also hold a privileged position in auditory language comprehension; listeners are faster to detect phonemes in stressed syllables [25], lexical access is more disrupted by the mispronunciation of stressed syllables than unstressed syllables [26], and listeners tend to interpret stressed syllables as word onsets [27,28]. Moreover, listeners use the pattern of strong and weak syllables to predict what words will come next [29], and to resolve lexical ambiguity [30,31,32,33].

Like speakers and listeners, there is evidence that readers are also sensitive to a metric structure. For example, readers spend more time fixating four-syllable words with two stressed syllables (e.g., RAdiAtion) than four-syllable words with one stressed syllable (e.g., geOmetry) [11]. In silent reading, syntactically ambiguous sentences are more likely to be resolved in ways that maintain alternating strong and weak syllables [14,15]. In the study that serves as the inspiration for the current study, Breen and Clifton tracked participants’ eye movements as they read limericks designed to induce readers to generate strong expectations about the stress pattern of upcoming words [13]. The target word in the critical items, which was always the final word of the second line of the limerick, was a stress-alternating noun–verb homograph; these words are realized with strong–weak (SW) stress as a noun (e.g., PERmit), but weak–strong (WS) stress as a verb (e.g., perMIT) [29]. In this way, the target was either SW or WS, and this lexical stress pattern was either consistent or inconsistent with the metric structure of the limerick (see Table 1). Throughout this paper, we will refer to the occurrence of an inconsistent SW word when a WS word is predicted as a strong–weak (SW) violation, and to the occurrence of an inconsistent WS word when a SW word is predicted as a weak–strong (WS) violation.

Breen and Clifton predicted that readers would encounter difficulty whenever the stress pattern of the target word mismatched the pattern of the limerick. However, they only observed an effect of metric mismatch for WS violations (e.g., Table 1D); reading times for SW violations (e.g., Table 1B) did not differ from those of consistent SW words. Breen and Clifton argued that these results reflect the uneven distribution of SW and WS words in the English lexicon; 85–90% of content words in English have an initial stressed syllable [34]. Specifically, there is a minimal cost to encountering a SW word in a context where a WS word is predicted because SW is the default stress pattern. Identifying a WS word in a context that predicts SW, on the other hand, is costly because of both the conflict with context and the lower base frequency of the WS pattern. This interpretation is supported by previous work showing that auditory word identification is more disrupted when a canonically SW word is pronounced as WS, than when a canonically WS word is pronounced as SW [35]. Moreover, the observed effect was not on initial reading times, but only on the combined duration of fixations on the target word and time spent rereading earlier sentence material. The latency of this effect, therefore, suggests that the WS violation did not disrupt initial reading times but required later reanalysis.

### 1.2. Event-Related Potential Studies of Explicit Linguistic Metric Processing

In ERP investigations of explicit metric processing during speech perception, multiple methods have been used to investigate metric violations. One major source of variation among these studies is whether the metric violation is determined by the lexical stress pattern of the word in isolation or only by the context in which the word occurs. In studies of the first variety, researchers presented multisyllabic words auditorily with the correct or incorrect stress pattern either in isolation [36,37,38] or in a sentence context [39,40]. In studies of the second variety, researchers established a context that created an expectation of a specific metric pattern, then presented a target that had the correct metric pattern in isolation but was consistent or inconsistent with the expected pattern created by the context. One such paradigm used word strings to create metric context: listeners heard a string of three or four prime words with the same lexical stress pattern (all SW or all WS, e.g., BANKer, HELPful, PARty or moRALE, emBRACE, deLIGHT) followed by a target word with the same stress pattern as the primes or the opposite pattern [41,42]. In another such paradigm, participants heard sentences with a consistent metric structure including a target which was either consistent or inconsistent with the established pattern [43,44,45,46,47,48] (e.g., stress clash in “The chamPAGNE COCKtails are very delicious”). A final method used cross-modal information to inform prosodic interpretation, as in [49] where participants viewed pictures which disambiguated the meaning of semantically ambiguous two-syllable strings like greenhouse, which are disambiguated by stress patterns (GREENhouse vs. green HOUSE).

Regardless of the type of manipulation, these ERP studies demonstrate that encountering metric violations while listening generally gives rise to an early negativity between 250 and 500 ms [36,37,38,39,40,41,42,43,44,45,46,47,48]. However, this early effect is not consistent across studies, in terms of timing and polarity. Some of the variance can be explained by the different responses to SW violations and WS violations in two-syllable words; SW violations, where a SW word appears when a WS word is predicted, typically elicit an early negativity [41,42,43,44,45,46,47,49]. The results are more mixed for WS violations, where a WS word appears when a SW word is predicted, which elicit an early negativity in some cases [41,42] but has also been shown to elicit an early positivity relative to predicted metric patterns [36,37,40,42,48]. In two studies, both SW and WS violations elicited an early negativity, but the negativity to SW violations peaked earlier [41,49].

Additionally, explicit metric violations have often been shown to elicit a late positivity between 500 and 1000 ms [36,37,38,39,40,42,43,44,46,48]. In contrast to the early time window, this later effect does not seem to differ in polarity or timing as a function of target lexical stress pattern. However, its presence is dependent on the experimental task; in cases where the participants’ task is to make an explicit assessment of the accuracy of the metric structure of the target, that target usually elicits a late positivity [36,37,38,46,48,49], though this is not always the case [45,47]. In contrast, if the participants’ task does not include a specific assessment of the metric structure, a late positivity is absent [41]. Indeed, in cases where the explicitness of a metric judgment is varied within the experiment, a late positivity is generally evident only when the task requires this judgment [39,40,42,43,44].

Despite some variation across studies, these neural effects of metric inconsistency appear to be distinct from the neural effects of either syntactic or semantic violations. Syntactic violations typically elicit a biphasic response consisting of a left-lateralized anterior negativity peaking around 300 ms (LAN) and a posterior positivity peaking around 600 ms after stimulus onset (P600/LPC) [50]. A simultaneous test of metric and syntactic violations reported distinct negativities for each violation type however, with the negativity to metric violations occurring earlier than the negativity evoked by syntactic violations (which was interpreted as a LAN) [43]. Semantic violations typically elicit a parietally-maximal negativity around 400 ms (N400) [51]. Although some authors have interpreted the early negativity elicited by metric violations as an N400 [39,40], this metric negativity has been observed in response to illegal stress shifts in pseudowords which have no lexico-semantic content and should not result in an N400 [45]. Further, semantic incongruity and metric incongruity have been shown to modulate the amplitude of an early negativity differently when considered in the same design, even by authors who categorize deviations from a predicted metric structure as N400 effects [39,40]. Finally, [44] observed that simultaneous metric and semantic violations lead to a larger negativity than that observed for semantic violation alone, and [52] used neuroimaging to demonstrate that the responses to semantic and metric violations have different neural generators, providing evidence that metric violations are not simply processed as semantic violations.

### 1.3. Event-Related Potential Studies of Implicit Linguistic Metric Processing

ERPs have also been used to explore implicit metric representations during silent reading. In one study, readers were presented with strings of four two-syllable English prime words with consistent lexical stress patterns, followed by a target word that was consistent or inconsistent with the stress pattern of the previous words [53]. Both SW and WS violations resulted in a larger fronto-central negativity from 250–400 ms after word onset, relative to words with a predicted stress pattern. In addition, all SW targets, whether consistent or inconsistent with the context, elicited a larger negativity (350–450 ms after word onset) than WS targets. In another study exploring silent metric processing in word lists, readers were presented with strings of three two-syllable German prime words followed by a SW or WS target. In this case, there were no observable ERP differences for SW violations, but WS violations were more positive than correct WS targets in three time windows: between 250–400, 400–600, and 600–800 ms after target onset [54]. A final study presented participants with an auditory tone sequence with a SW or WS pattern followed by a visually presented two-syllable English word which was consistent or inconsistent with the tone sequence stress pattern [55]. The results demonstrated a larger negativity from 300–700 ms after target presentation for SW violations compared to correct SW targets, but no significant ERP effect for WS violations. In general, these studies demonstrate that, similar to explicit metric violations, implicit metric violations often evoke an early negativity that is more reliably observed for SW than WS violations. Moreover, two of these studies are consistent with results from explicit meter studies in that when the task does not require an explicit metric judgment (and none of these did; rather, participants’ task was to make an old/new judgment of the target [53], a lexical decision judgment [55], or answer a semantic question about the word strings [54]), there is no late positivity.

Multiple factors could be contributing to the variability in results observed across previous investigations of ERP responses to implicit metric violations. First, these studies have used different target words in the SW and WS conditions, meaning that the observed results may reflect differences beyond prosody, including phonetic, orthographic, or lexical differences between conditions. Second, these studies used single words or word lists to create metric expectations but, in these contexts, readers are not required to fully process the syntactic and semantic structure of the targets; this variability could lead to heterogenous depth of processing across conditions. Therefore, in the current study, we implemented metric expectations using metrically regular rhyming couplets, which encourage readers to make strong predictions about when strong and weak syllables will occur but also require deep linguistic processing. Moreover, our target words are stress-alternating noun–verb homographs, which can have SW or WS stress depending on the syntactic category. In this way, readers are exposed to the same visual, orthographic, and segmental input across all conditions.

If readers are generating implicit metric predictions during silent reading, we predict that targets which are inconsistent with the metric context will result in early differences in the ERP waveform compared to metrically consistent targets. However, based on prior work, we predict that this early effect may differ depending on the type of violation. Specifically, we predict SW violations will elicit an early negativity relative to consistent SW words. Conversely, WS violations may result in either a reduced negativity, or a positivity, relative to WS consistent targets. Moreover, we predict the absence of a late positivity in response to metric violations, as participants are not making explicit judgments about the metric structure.

## 2. Materials and Methods

### 2.1. Participants

Eighteen participants from Mount Holyoke College with an average age of 20 years (SD = 1.57 years) contributed data to the analyses. Seventeen participants identified as female and one identified as nonbinary/genderqueer. All participants were right-handed native speakers of American English, meaning they had been speaking English in the US since at least the age of three. One participant was born outside the US to English-speaking parents and moved to the US at age three. Five participants identified as bilingual as they had acquired high proficiency in another language starting before the age of three. All participants reported having normal or corrected-to-normal vision and had not taken psychoactive medications in the 24 h prior to the experiment. For the two-hour experiment, participants received compensation in the form of Psychology course research credit or $20. Data were collected but discarded from an additional four female participants due to voluntary withdrawal from the experiment (*n* = 1), recording equipment malfunction (*n* = 1), or excessive noise in the EEG (exclusion of more than 50% of trials from one or more conditions due to artifact; *n* = 2).

### 2.2. Materials

Experimental materials consisted of 160 limerick couplets (i.e., the first two lines of the limerick) adapted from the stimuli in [13], in which the final word of the second line was one of 40 stress-alternating noun–verb homograph targets (see Table 1). The stress pattern of these targets varies depending on the syntactic category: the noun form has a strong–weak pattern (e.g., PERmit), the verb (or adjective) form has a weak–strong pattern (e.g., perMIT). The homographs were selected from [29] and the Kucera–Francis corpus [56]. The frequency of occurrence of each target as a noun or verb/adjective in the Kucera–Francis corpus did not differ, as measured by a paired *t*-test, *t*(39) = 0.28, *p* = 0.78.

For each target homograph, four couplets were constructed, crossing the factors stress pattern (SW vs. WS) and metric consistency (consistent vs. inconsistent). All experimental couplets can be found in the Appendix A. The first line of each couplet established the metric and rhyming context for the target word. The stress pattern manipulation was implemented such that for half of the couplets, the target (e.g., permit in Table 1) was the noun form with a SW pattern (Table 1A,B). For the other half, the target was the verb/adjective form (Table 1C,D). The metric consistency manipulation meant that for half of the couplets, the stress pattern of the target homograph was consistent with the stress pattern predicted by the couplet (Table 1A,C). For the other half, the stress pattern of the target was inconsistent with the established pattern (Table 1B,D). The occurrence of an inconsistent SW word when a WS word is predicted is a strong–weak (SW) violation (Table 1B)*,* and the occurrence of an inconsistent WS word when a SW word is predicted is a weak–strong (WS) violation (Table 1D).

In addition to the 160 experimental couplets, participants read 160 filler couplets which were always metrically consistent but varied in the stress pattern of the target regions (see Table S1 and examples (1), (2)). In this way, participants read a total of 80 rhythmically inconsistent items in a pool of 320 (25% of the total). 

Examples: 

1. There once was a man from Peru, who dreamt about eating his **shoe**

2. There once was a young man named Randy, who loved to eat all kinds of **candy**

### 2.3. Procedure

**Figure 1:**
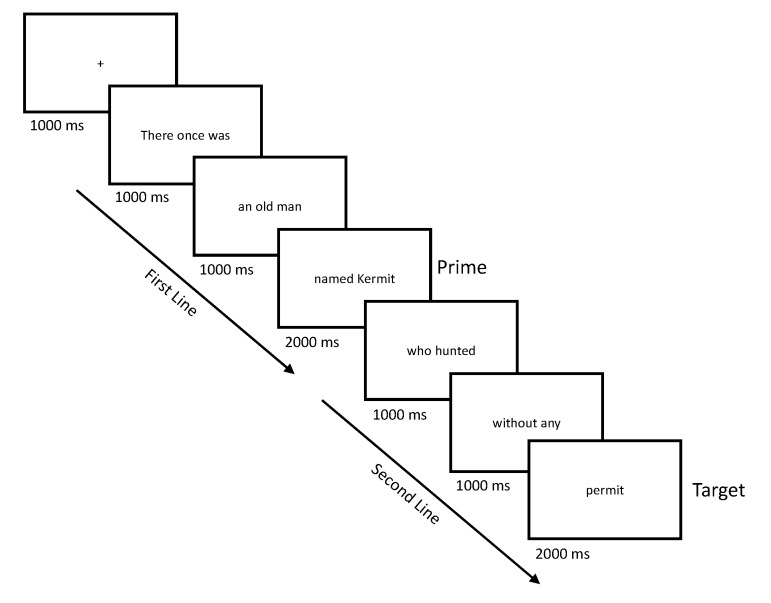
Presentation times in milliseconds of each region of the limerick couplets.

After providing informed consent, participants were seated comfortably in a sound-isolated room where they viewed the couplets on a computer screen located approximately 90 cm away. The 320 experimental and filler couplets were presented in a different randomized order for each participant. Each trial began with the presentation of the word “Ready?” which stayed on the screen until the participant responded with a keypress. The word was then replaced by a fixation cross, which remained on the screen for 1000 ms (Figure 1). Following the fixation cross, couplets were presented in six one-to-four-word (one-to-five-syllable) segments in the center of the screen (see Table S1). The 1st, 2nd, 4th and 5th segments were presented for 1000 ms each; the 3rd and 6th segments, corresponding to the end of the first and second lines, respectively, were presented for 2000 ms each. In the experimental couplets, the 3rd and 6th segments always contained two-to-three syllables, one of which was strong. The 1st, 2nd, 4th and 5th segments were more variable, but constrained so that each contained one strong syllable, and one-to-four weak syllables. The number of words and syllables varied across these segments because the couplets varied widely in terms of the number of words and stress patterns of the words that made them up. However, segments were consistently defined based on the first author’s intuition of natural syntactic and prosodic breaks in limerick structure.

To ensure that participants were reading for meaning, 25% of the filler trials (12.5% of all trials) were followed by a yes/no comprehension question about the semantic content. Participants held a response box in their lap for the duration of the experiment which they used to answer comprehension questions, and to advance the presentation of trials. Participants were given breaks between trials to allow time for blinking, as well as a longer break after every 40 trials; the length of these breaks were determined by the participant. The entire experimental session lasted approximately 2 h. All experimental procedures were approved in advance by the Institutional Review Board of Mount Holyoke College.

Reference-free electroencephalogram (EEG) data were collected using 64 active Ag/AgCl electrodes placed in an elastic cap and connected to a BioSemi Active-Two system, which digitized the EEG at a sampling rate of 2048 Hz and employed a hardware lowpass filter reaching −3 dB at 409.6 Hz. Reference-free EEG was also collected from two active electrodes attached bilaterally to the participant’s mastoids, and from four active electrodes placed above and below the left eye and bilaterally outside the outer canthi. All electrode offsets were brought below 20 mV at the start of the recording and kept below 50 mV throughout the recording. Continuous EEG data were referenced offline to the averaged mastoid recording, downsampled to 512 Hz, and filtered at 60 Hz using a Parks–McClellan notch filter. Bipolar vertical and horizontal electrooculogram (VEOG, HEOG) signals were derived by subtracting the above eye signal from the below eye signal, and the left from the right eye signal, respectively. Continuous EEG was segmented into epochs from 100 ms prior to target word onset to 800 ms following target word onset, and baseline-corrected to the 100 ms prestimulus period. Electrodes Oz and Iz were each identified as unusable for at least one participant and were excluded from further processing and analysis. Epochs containing eyeblinks or eye movements were identified algorithmically using moving window peak-to-peak voltage deflection detection on the VEOG channel (threshold = 150 µV, window size = 200 ms, window step = 25 ms) and step-like artifact detection on the HEOG channel (threshold = 100 µV, window size = 400 ms, window step = 25 ms), respectively. Additionally, epochs exceeding ±170 µV in any EEG channel were marked as artifact. The results of automatic artifact detection were then manually inspected and if needed, adjusted, and trials found to contain artifacts were excised. Artifact-free trials were then averaged by participant and condition; participants included in the analysis contributed data from at least 20 out of 40 trials (M = 31; SD = 6) in every condition. EEG data processing was performed in MATLAB using the EEGLAB [57] and ERPLAB [58] analysis packages.

### 2.4. Analysis

Previous ERP investigations of implicit linguistic metric processing have revealed effects across multiple time windows, with inconsistent time windows observed across studies [36,37,38,39,40,41,42,43,44,45,46,47,48,49]. We therefore opted to define our temporal regions of interest using a data-driven approach. To minimize implicit multiple comparisons when selecting time windows [59], we performed a series of cluster-based permutation tests over a moving 50 ms window (5 ms step) using the Mass Univariate ERP Toolbox [60]. These tests were performed separately for SW and WS violations. Within each moving window, electrodes at which the inconsistent vs. consistent *t*-test of mean window amplitude resulted in *p* ≤ 0.01 were identified, then clustered if they were within 5.44 cm of one another. Cluster magnitudes were then calculated as the sum of all *t*-scores for electrodes contained within a cluster. Lower-tailed *t*-tests were used for the SW comparisons based on prior findings that SW violations consistently elicit relative negativities [36,41,44,45,49,53,55], whereas two-tailed *t*-tests were used for the WS comparisons based on prior findings that WS violations elicit both relative negativities and positivities [40,41,42,54]. This process was replicated over 5000 shuffled iterations, and a cluster magnitude threshold was defined as the magnitude that clusters met or exceeded on only 5% of the shuffled (i.e., chance) iterations. Moving windows within which any clusters identified in the experimental data met or exceeded the cluster magnitude threshold were defined as temporal regions of interest (see Figure S1). This approach revealed three regions of interest, which were further investigated using conventional, ANOVA-based ERP analyses: 80–155 ms (SW), 325–375 ms (SW), and 365–435 ms (WS).

**Figure 2:**
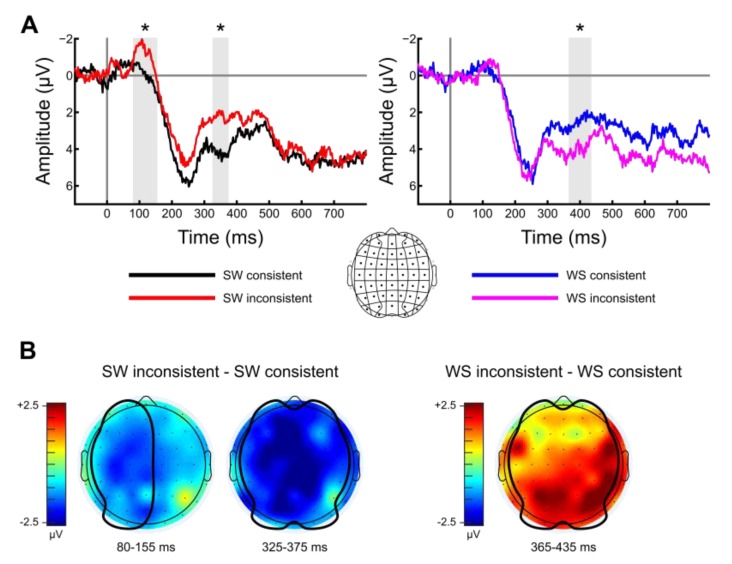
Event-related potential (ERP) results. (**A**) Effects of metric predictability on a grand average (*n* = 18) waveform amplitude for strong–weak (SW) targets (left) and weak–strong (WS) targets (right). Temporal regions of interest identified in the cluster-based permutation analyses are highlighted in grey. Temporal regions of interest that revealed a significant (*p* < 0.05) main effect of metric consistency in conventional ANOVA analyses are indicated with an asterisk. Waveforms are averaged over the 49 electrodes included in the ANOVA analyses; the 7 (anteriority) × 7 (laterality) grid arrangement used to model electrode position in all ANOVAs is shown in the inset. (**B**) Scalp maps showing the topography of mean amplitude differences between the inconsistent and consistent conditions within the two temporal regions of interest identified for SW targets (left), and the one temporal region of interest identified for WS targets (right). The scalp region over which a significant (*p* < 0.05) main effect of metric consistency was observed within the specified time window is outlined in black for each scalp map.

We selected 49 electrodes for conventional, ANOVA-based ERP analysis (Figure 2). Scalp position was treated as two factors in the statistical model: electrode anteriority had seven levels ranging from most anterior to most posterior electrodes, and electrode laterality had seven levels, ranging from left to right. Based on our cluster-based permutation tests, we assessed SW ERP amplitudes in two time windows (80–155 ms and 325–375 ms), and WS ERP amplitudes in one time window (365–435 ms). Mean amplitudes from each participant in each time window were entered into a 2 (metric consistency) × 7 (anteriority) × 7 (laterality) repeated-measures ANOVA. Significant and marginal interactions of metric consistency with electrode position in the absence of a main effect of metric consistency were further investigated with follow-up ANOVAs over constrained scalp regions. Only main effects and interactions which involve metric consistency will be discussed. Whenever Mauchly’s Test indicated that the assumption of sphericity had been violated for comparisons with more than two levels, Huynh–Feldt-corrected degrees of freedom were used to compute statistical significance. All statistical analyses were implemented in the R statistical framework [61] with the ez package [62].

## 3. Results

### 3.1. Behavioral

Participants answered comprehension questions with an average accuracy rate of 96.25% (SD = 4.2%), demonstrating that they were attending to the couplets and engaged with the task.

### 3.2. Event-Related Potentials

#### 3.2.1. SW Violations

SW violations elicited a negativity from 80–155 ms relative to predicted SW targets over left and medial scalp positions (Figure 2). An overall 2 × 7 × 7 ANOVA looking only at SW targets revealed a marginal interaction of metric consistency and electrode laterality, *F*(2.74,46.55) = 2.38, *p* = 0.087, *η*^2^ = 0.007. A follow-up 2 × 7 ANOVA looking only at SW targets over left and medial scalp positions revealed a negativity elicited by SW violations relative to predicted SW targets, *F*(1,17) = 4.75, *p* = 0.044, *η*^2^ = 0.14.

SW violations also elicited a negativity from 325–375 ms relative to predicted SW targets over the entire scalp, that was largest over left and medial scalp positions (Figure 2). An overall 2 × 7 × 7 ANOVA looking only at SW targets revealed a main effect of metric consistency, *F*(1,17) = 5.32, *p* = 0.034, *η*^2^ = 0.09, and a marginal interaction of metric consistency and electrode laterality indicated that this effect was largest over left and medial scalp positions, *F*(3.38,57.45) = 2.51, *p* = 0.06, *η*^2^ = 0.004.

#### 3.2.2. WS violations

WS violations elicited a positivity from 365–435 ms relative to predicted WS targets over the entire scalp, that was largest over central and posterior scalp positions (Figure 2). An overall 2 × 7 × 7 ANOVA looking only at WS targets revealed a main effect of metric consistency, *F*(1,17) = 4.99, *p* = 0.039, *η*^2^ = 0.06, and a marginal interaction of metric consistency and electrode anteriority indicated that this effect was largest over central and posterior scalp positions, *F*(2.68,45.54) = 2.78, *p* = 0.06, *η*^2^ = 0.01.

## 4. Discussion

The goal of the current study was to investigate the realization of metric representations during silent reading using ERPs. Participants silently read metrically regular rhyming couplets in which the final target word had a strong–weak (SW) or weak–strong (WS) lexical stress pattern that was either consistent or inconsistent with the metric stress pattern predicted by the couplet. The results demonstrated that SW targets which were inconsistent with the stress pattern of the couplet (i.e., SW violations) elicited two separate negativities (80–155 ms and 325–375 ms after word onset) relative to SW targets which were consistent with the predicted stress pattern. Conversely, WS targets inconsistent with the stress pattern of the couplet (i.e., WS violations) elicited an early positivity (365–435 ms after word onset) relative to WS targets which were consistent with the predicted stress pattern. Neither SW nor WS violations elicited a late positivity. Together with prior results, the current results support the Implicit Prosody Hypothesis, which maintains that readers are generating implicit versions of prosodic structure even when reading silently, and that these representations are similar to explicit ones.

The observation of a significant negative left-lateralized deflection from 80–155 ms in response to SW violations is an unexpected result based on prior work on explicit and implicit linguistic metric processing. Few studies of linguistic meter have reported consistent differences in components this early, though one study demonstrated a significant negativity between 100–320 ms in response to an inappropriate stressed syllable [46]. However, negativities in the 100–200 ms time window have been widely observed in response to metric violations in musical studies. This effect, termed the metric mismatch negativity (MMN), has been observed when a strong tone occurs at an unpredicted temporal location (i.e., when a weak tone is predicted) [63,64,65]. This situation is analogous to the circumstance under which we observed the early negativity in the current study, such that a strong beat at a predicted weak time elicits the early negativity (SW violation), whereas a weak beat at a predicted strong time does not (WS violation). Importantly, as this effect was detected based on a marginal interaction of metric consistency with electrode position and this is the first study we are aware of to report this early negativity in response to an implicit strong beat occurring at a predicted weak time, additional experiments will be required to determine the reliability and meaning of this component.

The negativity between 325–375 ms observed for SW violations is consistent with results from previous investigations of both explicit and implicit violations of metric structure. Specifically, previous studies have demonstrated that SW metric violations result in a negative deflection in the 250–500 ms range relative to metrically consistent targets [36,41,44,45,49]. Moreover, a similar effect has also been shown in a small set of studies investigating metric structure in silent reading of single words [53,55]. The current study extends this finding to silent reading of metric violations in sentence contexts using orthographically identical items across all conditions. The observation of a positivity for WS violations from 365–435 ms after word onset is also consistent with both prior listening and reading studies. Two prior studies of explicit metric violations [40,42] and one prior study of implicit metric processing [54] have observed positive deflections for consistent WS targets relative to inconsistent WS targets. Our results therefore suggest that prior findings of different responses to SW and WS violations are not simply due to idiosyncratic differences between the SW and WS target items chosen for these prior experiments, but do indeed reflect the activation of abstract metric representations during silent reading.

The different results observed across multiple studies for SW vs. WS violations may be due to differences in the underlying phonological structure of the target words. According to [17], the trochaic foot (SW) is the default phonological structure in Germanic languages, including English. This phonological constraint is realized in the lexical stress patterns of words, such that most two-syllable words begin with a stressed syllable (85–90% of the time in English [34]; 73% of the time in German [66]). This asymmetry means that accessing a SW (trochaic) representation of a target is globally easier than accessing a WS (iambic) representation, irrespective of the context in which the target occurs. Therefore, the lexical representation of a SW target is harder to access when its stress pattern conflicts with the local metric context, than when its stress pattern is consistent with the local context. Conversely, resolving WS violations is more challenging for readers, due to conflicting cues in both the local environment and the global environment.

Under this view of phonological asymmetry, the negativity observed between 325–375 ms for SW violations in the current study, and in a similar time window in other studies, could be related to the N400, which reflects the ease with which lexical access is achieved. The negativity for SW violations could reflect either additional lexical processing due to the added difficulty of accessing the appropriate lexical content in the presence of lexical stress mismatch, or lexical repair processes due to automatic activation of the metrically consistent, but semantically inconsistent, alternate form of the noun/verb homograph. However, it is important to note that this interpretation of the negativity as indexing lexical processing is challenged by previous work exploring simultaneous violations of metric and semantic structure, in which the latency and distribution of the negativity differs across violation types [39,40], as well as evidence that metrically inconsistent pseudowords also elicit such negativities, even though they lack semantic context [45]. Alternatively, it could be that the negativity we observed in the current experiment indicates the violation of a consistent, rule-based sequence, in this case realized as the metric structure [45].

In contrast, the positivity observed between 365–435 ms for WS violations in the current study, and in a similar time window in other studies, could be related to conflict processing. When a WS violation occurs, the reader must resolve the conflict between a metric context which leads them to predict a SW target and a semantic context which leads them to predict a WS target. In addition, there is the added conflict that WS two-syllable words are phonologically marked in the language. These factors together may lead to the observed positivity, which is signaling an error in processing that is harder for readers to recover from. This interpretation is consistent with previous ERP research of the metric structure in German, where metric violations in three-syllable words that did not violate metric foot structure led to an early negativity, whereas violations that also conflicted with foot structure resulted in an early positivity [36], similar to the results in the current study.

Consistent with other explicit and implicit metric processing studies that do not involve an explicit metric task, we did not observe evidence of a late positivity for metric violations relative to consistent metric conditions. Previous studies of both explicit and implicit metric processing demonstrate that late positivities in response to metric violation are most likely observed when the participant’s task is to assess the metric structure. Indeed, only one previous study of implicit metric processing observed a late positivity in response to metric violations [54] while two others did not [53,55], and none of these studies required an explicit metric judgment. This interpretation is in line with previous work showing a dissociation between early and late ERP effects of syntactic violations, where early negativities are thought to reflect automatic processing and late positivities are thought to reflect controlled processes of repair [67,68] and the difficulty of the required repair process [69]. The current results suggest that although both implicit and explicit metric violations are automatically detected, as evidenced by early (<500 ms) waveform differences, only violations that rise to the level of awareness give rise to a late positivity.

It is also possible that the lack of a late positivity in the current study reflects a lack of power; our choice to present the same orthographic information across conditions meant that the number of items in the experiment was limited by the number of two-syllable stress-alternating noun–verb homographs in English that were known to our participants and could be embedded in rhyming couplets. Moreover, compared to previous studies using word lists, the stress pattern of the target in the current study was locally ambiguous, and only disambiguated by the implicit metric structure provided by the context. Although this manipulation is a better test of the abstract metric structure compared to other studies that used different items across SW and WS conditions, it produces a less clearly defined metric violation than paradigms employing single target words with unambiguous stress patterns.

Although the current results are generally consistent with prior ERP work on explicit and implicit linguistic metric structures, they are inconsistent with results observed in a previous eye-tracking experiment using the same materials. Recall that Breen and Clifton observed inflated reading times only for WS violations, and not for SW violations [13]; moreover, these effects were observed only in relatively later reading time measures. Conversely, our results demonstrate significant early ERP differences for both SW and WS violations, though they differ in polarity, timing, and topography. These differential effects are likely due to differences in the temporal control of stimulus presentation between the studies. In Breen and Clifton’s experiment, participants read normally at their own pace, meaning they could take as much time as needed to process material in advance of the critical word, and could look back to prior sentence material to resolve difficulty generated at the target word. In contrast, materials in the current study were presented in a region-by-region segmented fashion, giving participants less time to generate predictions about upcoming material, and disallowing regressions. Moreover, the fact that the current materials were presented in a time-controlled manner means that the metric structure of the sentence materials was more obvious for readers, making the metric inconsistency more explicit, resulting in significant ERP effects of both types of metric violations.

Future work could directly investigate the role of temporal stimulus control on implicit metric violation processing by replicating the current paradigm using simultaneous collection of eye-tracking data and ERPs, a method which has already been used to successfully adjudicate debates about linguistic processing in eye movements [70,71]. In this way, the role of metric inconsistency in silent reading could be assessed without explicitly controlling the timing of materials. Additionally, while current results demonstrate that readers engage in implicit prosody during silent reading of poetry, it is an open question to what extent these findings generalize to normal reading. The couplets used in the current study were designed to have strict metric and rhyming structure, which is rare in non-poetic language. However, our study does provide an insight to the role of meter in implicit prosody. To determine whether our result can be replicated in non-poetic contexts which do not have concomitantly high metrical expectancies, future work will explore differences in brain activity in response to metric violations in silently-read prose sentences.

## 5. Conclusions

The current results provide further evidence of an intimate link between metric processing during listening and metric processing during silent reading, which may help inform our understanding of previously described relationships between children’s sensitivity to an auditory metric structure and silent reading comprehension. For example, the ability of older children to track a perceived metric structure predicts phonological awareness and reading outcomes [72,73], and children’s ability to detect a mis-stressed word predicts phonological awareness and word knowledge [74]. It may be the case that these reading abilities are facilitated by implicit metric structure representations. This claim is further bolstered by a relationship between prosodic fluency and reading comprehension in high school students—those who demonstrate higher prosodic fluency also showed an increased comprehension ability [75,76]. Research about implicit prosody and the underlying neurocognitive processes occurring during silent reading may, therefore, inform future work designing prosodic interventions to improve children’s reading comprehension abilities.

## Figures and Tables

**Table 1:** Metric structure of experimental couplets in each of the four conditions for the target word ‘permit’.

		W	S	W	W	S	W	W	S	W
A.	Strong–weak, consistent	There	*once*	was	an	*old*	man	named	*Ker-*	mit
		who	*hunt-*	ed	with-	*out*	an-	y	***PER-***	**mit**
B.	Strong–weak, inconsistent	There	*once*	was	an	*old*	man	named	*Britt*	
		who	*hunt-*	ed	with-	*out*	a	*PER-	***mit***	
C.	Weak–strong, consistent	There	*once*	was	an	*old*	man	named	*Britt*	
		whose	*vic-*	es	no	*wife*	could	**per-**	***MIT***	
D.	Weak–strong, inconsistent	There	*once*	was	an	*old*	man	named	*Ker-*	mit
		Whose	*gamb-*	ling	his	*wife*	would	not	*per-	**MIT**

Italics and underlines indicate metrically strong syllables, bold indicates target words, and capital letters indicate lexical stressed syllables within target words. Asterisks (*) indicate metrically inconsistent targets. Screen breaks are indicated with solid vertical lines. Text emphasis is for descriptive purposes only; in the experiment, all words were presented in plain text (see Figure 1).

## Supplementary Materials

The following are available online at https://www.mdpi.com/2076-3425/9/8/192/s1, Table S1: Distribution of stress patterns across segments for experimental and filler couplets, Figure S1: Moving window cluster-based permutation test results.

## Appendix A

1a. SW/consistent
You must hear/my story,/your highness. I have the/young princess’s/address

1b. SW/inconsistent
My workspace/is such a/big mess. I lost an/important/address

1c. WS/consistent
My workspace/is such a/big mess. My clutter/I have to/address

1d. WS/inconsistent
You must hear/my story,/your highness. Your habits I/find I must/address

2a. SW/consistent
The guy who/got lost on/a flyby Dropped all of/his bombs on an/ally

2b. SW/inconsistent
I know a/young woman/from Rye, Who’d make such/a lovely/ally

2c. WS/consistent
I know a/young woman/from Rye, With whom I/would like to/ally

2d. WS/inconsistent
The guy who/got lost on/a flyby Killed folks with whom/we want to/ally

3a. SW/consistent
I just saw/a dog and/a tomcat, Engaged in/some furious/combat

3b. SW/inconsistent
I witnessed/a dog and/a cat, Engaged in/some angry/combat

3c. WS/consistent
I witnessed/a dog and/a cat, Who seemingly/tried to/combat

3d. WS/inconsistent
I just saw/a dog and/a tomcat, That we must/be ready to/combat

4a. SW/consistent
I heard someone/say through/the grapevine: The farmer is/driving his/combine

4b. SW/inconsistent
The farmer/got caught/drinking wine, Then harvesting/in his/combine

4c. WS/consistent
The farmer/got caught/drinking wine, And shotguns/and booze don’t/combine

4d. WS/inconsistent
I heard someone/say through/the grapevine: That farmer is/hoping to/combine

5a. SW/consistent
I processed/some prints in/the darkroom Of people I’d/met on a/commune

5b. SW/inconsistent
I know some/who worship/the moon, And live/in a hippie/commune

5c. WS/consistent
I know some/who worship/the moon, With nature/they like to/commune

5d. WS/inconsistent
I processed/some prints in/the darkroom Of folks who/just wanted to/commune

6a. SW/consistent
If out in/the mountains/you backpack, Your team must/agree to this/compact

6b. SW/inconsistent
Before you/head out with/that pack, Your team has/to sign this/compact

6c. WS/consistent
Before you/head out with/that pack, Be sure that/your gear is/compact

6d. WS/inconsistent
If out in/the mountains/you backpack, Your gear must/be basic and/compact

7a. SW/consistent
The crew worked/so hard for/their paychecks They thought they’d/develop a/complex

7b. SW/inconsistent
There once was/a young man/named Rex Who owned/an apartment/complex

7c. WS/consistent
There once was/a young man/named Rex Whose theories/were big and/complex

7d. WS/inconsistent
The crew worked/so hard for/their paychecks Their work was/so terribly/complex

8a. SW/consistent
We stayed in/the woods at/a campground, Which wasn’t too/far from a/compound

8b. SW/inconsistent
We got that/old dog at/the pound He came from/a private/compound

8c. WS/consistent
We stayed in/the woods at/a campground, Our pleasure in/nature to/compound

8d. WS/inconsistent
We got that/old dog at/the pound Our sadness/will surely/compound

9a. SW/consistent
There was a/young heroin/addict, Who ended up/causing a/conflict

9b. SW/inconsistent
My parents/are being/quite strict. Our views are/in open/conflict

9c. WS/consistent
My parents/are being/quite strict. Their wishes/and mine do/conflict

9d. WS/inconsistent
There was a/young heroin/addict, Whose habits and/others did/conflict

10a. SW/consistent
The athlete/who just failed/a drugtest, Will soon face/a challenging/contest

10b. SW/inconsistent
The athlete/who thinks he’s/the best Just lost an/important/contest

10c. WS/consistent
The athlete/who thinks he’s/the best Holds titles/that others/contest

10d. WS/inconsistent
The athlete/who just failed/a drugtest, Is planning the/charges to/contest

11a. SW/consistent
Although that/young man is/an addict, He really should/not be a/convict

11b. SW/inconsistent
I think that/the judge was/too strict In sentencing/that young/convict

11c. WS/consistent
I think that/the judge was/too strict, The jury too/quick to/convict

11d. WS/inconsistent
Although that/young man is/an addict, I think that the/judge shouldn’t/convict

12a. SW/consistent
That man applies/way too much/hair grease. A friend should/suggest a big/decrease

12b. SW/inconsistent
Forgive me/for stating/my peace, But you must/commence a/decrease

12c. WS/consistent
Forgive me/for stating/my peace, Your appetite/you must/decrease

12d. WS/inconsistent
That man applies/way too much/hair grease. I think the/amount he should/decrease

13a. SW/consistent
The Soviet/spy is a/suspect. The case has/but one major/defect

13b. SW/inconsistent
The Soviet/spy they/suspect, Has plans with/a major/defect

13c. WS/consistent
The Soviet/spy they/suspect, Is planning/quite soon to/defect

13d. WS/inconsistent
The Soviet/spy is/a suspect. I heard that/he’s planning to/defect

14a. SW/consistent
In nothing/but jeans and/a t-shirt, That man took/a trip ‘cross the/desert

14b. SW/inconsistent
The fighting/he tried/to avert, By running off/through the/desert

14c. WS/consistent
The fighting/he tried to/avert, By choosing/his squad to/desert

14d. WS/inconsistent
In nothing but/jeans and a/t-shirt, A soldier his/squad chose to/desert

15a. SW/consistent
I know of/an elegant/female Her outfits lack/no fashion/detail

15b. SW/inconsistent
There once was/a woman/named Gail Whose fashion/had every/detail

15c. WS/consistent
I know of/an elegant/female Who wanted/her auto to/detail

15d. WS/inconsistent
There once was/a woman/named Gail Who wanted/her car to/detail

16a. SW/consistent
We once had/a tiresome/house guest, Who loved to/read Birdwatcher’s/Digest

16b. SW/inconsistent
We once had/a friend as/a guest, Who loved to/skim Reader’s/Digest

16c. WS/consistent
We once had/a friend as/a guest, Whose cooking/we could not/digest

16d. WS/inconsistent
We once had/a tiresome/house guest, Whose humor/was painful to/digest

17a. SW/consistent
The gymnast/requested/a recount Her score, she/thought, rated no/discount

17b. SW/inconsistent
He could not/afford the/amount, And asked for/a modest/discount

17c. WS/consistent
He could not/afford the/amount. The invoice they/would not/discount

17d. WS/inconsistent
The gymnast/requested/a recount She thought it/was wrongful to/discount

18a. SW/consistent
In order to/prove your/attendance You’ll have to/check in at the/entrance

18b. SW/inconsistent
This gorgeous/young woman/from France Made everyone/jam the/entrance

18c. WS/consistent
This gorgeous/young woman/from France Would often/the young men/entrance

18d. WS/inconsistent
There was a/young woman/whose nude dance Would always/the gentlemen/entrance

19a. SW/consistent
He tried not/to get badly/sidetracked. He needed/some raspberry/extract

19b. SW/inconsistent
The recipe/seemed quite/exact. It called for/some almond/extract

19c. WS/consistent
The recipe/seemed quite/exact. Some essence/you had to/extract

19d. WS/inconsistent
He tried not/to get badly/sidetracked Some essence/he wanted to/extract

20a. SW/consistent
The city/must safeguard/the seaports, To save us/from dangerous/imports

20b. SW/inconsistent
The panel/is set to/report On how much/we pay for/imports

20c. WS/consistent
The panel/is set to/report On how much/the city/imports

20d. WS/inconsistent
The city/must safeguard/the seaports, Because of how/much it now/imports

21a. SW/consistent
The man who/asked you for/a consult Was given/a horrible/insult

21b. WS/consistent
That woman/who likes the/occult, Is very/unsafe to/insult

21c. WS/inconsistent
The man who/asked you for/a consult Is no-one/you wanted to/insult

21d. SW/inconsistent
That woman/who likes/the occult, Will tolerate/no more/insults

22a. SW/consistent
The teacher/assigned them/a project To find an/unusual/object

22b. SW/inconsistent
The winners/will get to/select A shiny/expensive/object

22c. WS/consistent
The mayor/that we might/elect Has views to/which others/object

22d. WS/inconsistent
The teacher/assigned them/a project That forced many/parents to/object

23a. SW/consistent
There once was/an old man/named Kermit, Who hunted/without any/permit

23b. SW/inconsistent
There once was/an old man/named Britt Who hunted/without a/permit

23c. WS/consistent
There once was/an old man/named Britt Whose vices/no wife could/permit

23d. WS/inconsistent
There once was/an old man/named Kermit Whose gambling his/wife would not/permit

24a. SW/consistent
I know of/an old man/named Herbert, Who’s known around/town as a/pervert

24b. SW/inconsistent
The nun/did her best/to convert A man whom/they call a/pervert

24c. WS/consistent
That nun/did her best/to convert Young kids who/the truth do/pervert

24d. WS/inconsistent
I know of/an old man/named Herbert Who always the/truth tries to/pervert

25a. SW/consistent
There once was/a penniless/peasant, Who couldn’t/afford a nice/present

25b. SW/inconsistent
There once was/a clever/young gent, Who bought for/his girl a/present

25c. WS/consistent
There once was/a clever/young gent, Who had a/nice talk to/present

25d. WS/inconsistent
There once was/a penniless/peasant, Who went to/his master to/present

26a. SW/consistent
He couldn’t/hide all of/his misdeeds, But made off/with all of the/proceeds

26b. SW/inconsistent
In light/of the man’s/dirty deeds, He won’t/receive any/proceeds

26c. WS/consistent
In light/of the man’s/dirty deeds, On Monday/his trial/proceeds

26d. WS/inconsistent
He couldn’t/hide all of/his misdeeds On Monday/his retrial/proceeds

27a. SW/consistent
There once was/a crusty old/recluse, Who grew the/most wonderful/produce

27b. SW/inconsistent
There simply/is no good/excuse For failing to/eat your/produce

27c. WS/consistent
There simply/is no good/excuse For failing/to work and/produce

27d. WS/inconsistent
There once was/a crusty/old recluse, Whose garden great/harvests would/produce

28a. SW/consistent
With all of/their time spent/at recess, The children/make no forward/progress

28b. SW/inconsistent
The efforts/at peace,/I confess, Are making/no forward/progress

28c. WS/consistent
The efforts/at peace,/I confess, Will simply/no longer/progress

28d. WS/inconsistent
With all of/their time spent/at recess, The children will/soon fail to/progress

29a. SW/consistent
I noticed/a ruinous/defect In part of/the candidate’s/project

29b. SW/inconsistent
The man we/will likely/elect Endorses/this wacky/project

29c. WS/consistent
The mayor that/folks will/elect According/to what polls/project

29d. WS/inconsistent
I noticed/a ruinous/defect In what that/new candidate/projects

30a. SW/consistent
There once was/a young man/named Ernest, Who sponsored/a violent/protest

30b. SW/inconsistent
They put the/man under/arrest For leading/an angry/protest

30c. WS/consistent
They put the/man under/arrest, And gave him/no time to/protest

30d. WS/inconsistent
There once was/a young man/named Ernest, Who rounded up/people to/protest

31a. SW/consistent
In a voice/that was piercing/and treble, The serfs were/inspired by a/rebel

31b. SW/inconsistent
The infantry/failed to/repel The followers/of the/rebel

31c. WS/consistent
The infantry/failed to/repel The fighters/who want to/rebel

31d. WS/inconsistent
In a voice/that was piercing/and treble, The leader urged/peasants to/rebel

32a. SW/consistent
That basketball/star’s like a/bloodhound. He seeks out/and catches each/rebound

32b. SW/inconsistent
The basketball/star turned/around, and caught an/amazing/rebound

32c. WS/consistent
The basketball/star turned/around, And watched for/the shot to/rebound

32d. WS/inconsistent
That basketball/star’s like a/bloodhound. He waits for/each jumpshot to/rebound

33a. SW/consistent
I met an/old friend who/played baseball, Who warned of/a new safety/recall

33b. SW/inconsistent
I met an/old friend at/the mall, Who warned of/a safety/recall

33c. WS/consistent
I met an/old friend at/the mall, Whose name I/just could not/recall

33d. WS/inconsistent
I met an/old friend who/played baseball, But what his/name was I can’t/recall

34a. SW/consistent
There once was/a young man/named Eckerd Who broke an old/pole-vaulting/record

34b. SW/inconsistent
The athlete/won quite an/award For breaking/the scoring/record

34c. WS/consistent
The athlete/won quite an/award The cameras/were there to/record

34d. WS/inconsistent
There once was/a young man/named Eckerd Whose pole-vaulting/feats they did/record

35a. SW/consistent
Last year I/created a/stock fund. And managed to/get a big/refund

35b. SW/inconsistent
I have to/admit I/am stunned, You didn’t/give me my/refund

35c. WS/consistent
I have to/admit I/am stunned, My payments/you will not/refund

35d. WS/inconsistent
Last year I/created a/stock fund. The fees they/would happily/refund

36a. SW/consistent
The judges must/all watch/the replay To find out which/team won the/relay

36b. SW/inconsistent
A messenger/came by/today To find out/who won the/relay

36c. WS/consistent
A messenger/came by/today; A message/he had to/relay

36d. WS/inconsistent
The judges must/all watch/the replay. Results to the/coach they will/relay

37a. SW/consistent
I read an/unusual/essay ‘Bout how they/conducted a/survey

37b. SW/inconsistent
A lovely/young woman/named Fay Was asked to/complete a/survey

37c. WS/consistent
A lovely/young woman/named Fay The future/she liked to/survey

37d. WS/inconsistent
I read an/unusual/essay Describing how/folks tried to/survey

38a. SW/consistent
The cops are/an interesting/subject They bullied their/most recent/suspect

38b. SW/inconsistent
The cops/didn’t try/to protect A recently/collared/suspect

38c. WS/consistent
The cops/didn’t try/to protect The people/they chose to/suspect

38d. WS/inconsistent
The cops are/an interesting/subject They bully/the people they/suspect

39a. SW/consistent
A striking young/woman named/Rembrandt, From Portugal,/she was a/transplant

39b. SW/inconsistent
A striking young/dame named/van Zandt, From Spain was/a recent/transplant

39c. WS/consistent
A striking young/dame named/van Zandt Had roses/she hoped to/transplant

39d. WS/inconsistent
A striking young/woman named/Rembrandt, Had roses she/wanted to/transplant

40a. SW/consistent
To get to/the local gym’s/squash court, You must take/municipal/transport

40b. SW/inconsistent
The mafia/tried to/extort The captain/of public/transport

40c. WS/consistent
The mafia/tried to/extort A man who/had tried to/transport

40d. WS/inconsistent
To get to/the local/gym’s squash court, Your gear should/be ready to/transport
