# Supplementary material for: Event-Related Potential Evidence of Implicit Metric Structure during Silent Reading
Source: Brain Sci. 2019 Aug 8;9(8):192. doi: 10.3390/brainsci9080192 (PMC6721353; doi:10.3390/brainsci9080192)
Supplement: Supplementary file 1 [file brainsci-09-00192-s001.zip › brainsci-563567-supplementary.pdf]

Table S1. Distribution of stress patterns occurring at each segment position across all 160 experimental couplets and 160 filler couplets.

Stress Pattern	Experimental Couplets						Filler Couplets					
	Segment						Segment					
	1	2	3	4	5	6	1	2	3	4	5	6
SW	0	18	28	0	12	80	1	7	5	0	4	34
WS	6	6	68	3	0	80	6	14	94	9	1	13
WSW	125	102	52	115	69	0	135	117	37	81	17	8
WSWW	27	17	0	42	44	0	6	9	1	38	77	1
WWS	2	6	12	0	0	0	5	5	12	11	1	1
WWSW	0	2	0	0	3	0	5	6	2	19	2	1
WWSWW	0	0	0	0	0	0	2	0	0	2	17	0
SWW	0	9	0	0	32	0	0	2	0	0	41	0
S	0	0	0	0	0	0	0	0	9	0	0	102

Segments correspond to regions depicted in Table 1 and Figure 1. S = Strong; W = Weak. All columns sum to 160.

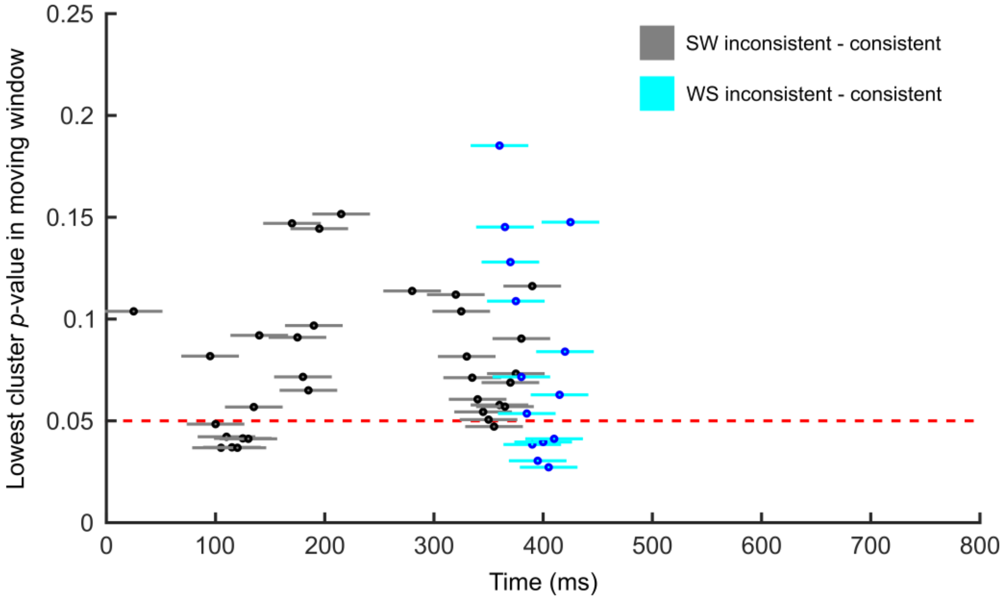

Figure S1. Moving window cluster-based permutation test results. Temporal region of interest selection was performed separately for each stress pattern (SW, WS) using a series of mass univariate cluster-based permutation tests comparing mean ERP amplitude for inconsistent vs. consistent targets in a 50 ms moving window (5 ms step). All window steps containing identified clusters are plotted as horizontal lines spanning their duration (with an open circle marking the step center), at a y-value indicating the lowest *p*-value calculated for any cluster in that window in the permutation test. See text for cluster inclusion thresholds and permutation test details; note also that no identified clusters contained more than one electrode. Cluster-containing window steps are plotted in grey for the SW stress pattern, and in cyan for the WS stress pattern. Permutation test *p*-values indicate for a given cluster the proportion of the shuffled (i.e., chance) iterations of the permutation test in which a cluster of equal or greater magnitude was identified. Any window step containing a cluster of magnitude observed in ≤ 5% of the shuffled iterations was considered a temporal region of interest. Collapsing across continuous and overlapping significant window steps, three temporal regions of interest were identified: 80–155 ms (SW), 325–375 ms (SW), and 365–435 ms (WS). No clusters were identified for either stress pattern beyond 500 ms after target word onset.
